# Comparison of Intestinal Microbiota of Blue Fox before and after Weaning

**DOI:** 10.3390/ani14020210

**Published:** 2024-01-08

**Authors:** Hang Su, Xinshuai Jiang, Hanyu Liu, Huixin Bai, Xiujuan Bai, Yuan Xu, Zhiheng Du

**Affiliations:** College of Animal Sciences and Technology, Northeast Agricultural University, Harbin 150030, China; suhang609@126.com (H.S.); s230501037@neau.edu.cn (X.J.); s230501039@neau.edu.cn (H.L.); bhxstriving@163.com (H.B.); bxiujuan630306@163.com (X.B.)

**Keywords:** weaning, blue fox, intestinal flora, 16s rRNA

## Abstract

**Simple Summary:**

The intestine is an important immune organ of the body in which the gut microbiota plays an important function. Gut microbiota is considered an important “organ” and is receiving increasing attention. This study investigated the diversity of gut microbiota in different intestinal segments of blue foxes, and the similarity between fecal microbiota and gut microbiota in samples taken before and after weaning. The types, distribution characteristics, and patterns of microorganisms in different intestinal segments of blue foxes differed before and after weaning. Except for the rectum, the dominant microbiota of each intestinal segment before and after weaning changed significantly. The fecal microbiota structure of weaned blue foxes cannot represent the exact structural characteristics of the entire gut microbiota but can represent the structural characteristics of the colon and rectum gut microbiota.

**Abstract:**

Intestinal flora plays an important role in maintaining the internal stability and health of the intestine. Currently, intestinal microbes are considered an important “organ” but are mostly ignored by people. This study evaluated the flora structure of each intestinal segment of blue foxes pre-weaning and explored the differences between the fecal flora and intestinal flora structure of each segment after weaning. Samples of intestinal contents from three blue foxes at 45 days of age (before weaning) and intestinal contents and feces samples from at 80 days (after weaning) were collected for 16s rRNA flora analysis. The species and distribution characteristics of microorganisms in different intestinal segments of blue foxes before and after weaning were different. Except for the rectum, the dominant flora of each intestinal segment of blue fox changed significantly after experiencing weaning, and the fecal flora structure of young fox at the weaning stage did not represent the whole intestinal flora structure but was highly similar to that of the colon and rectum. To sum up, the intestinal flora of blue foxes changed systematically before and after weaning. When performing non-invasive experiments, the microflora structure of the colon and rectum of blue foxes can be predicted by collecting fecal samples.

## 1. Introduction

The intestine is the key interface between animals and bacteria in the body. The microbiota resides on the surface of the intestinal mucosa and facilitates several functions, including providing nutrition, metabolism, and immunity [1]: (1) producing digestive enzymes such as amylase and cellulase [2]; (2) promoting digestion and absorption of nutrients [3]; (3) digesting and fermenting food fibers, generating short chain fatty acids, and providing energy for intestinal cells [4]; (4) releasing glucagon like peptide and participation in maintaining energy balance [5]; (5) contacting with gut symbiotic bacteria and foreign passing bacteria to create a wide range of immune barriers for the host [6]; (6) stimulating the maturation and cellular immune response of the host immune system. The resultant intestinal mucosal barrier can block food and most antigens in the intestine and participate in inducing immune tolerance [7], and (7) colonization, reproduction, and adherence to intestinal mucosa or epithelial cells in the host form a “biofilm barrier”, reducing the colonization of pathogenic microorganisms in the intestine and resisting the invasion of foreign pathogens [6].

Weaning is an important factor influencing changes in the intestinal flora of early mammals. During the weaning period in mammals, the physiological functions of the digestive and immune systems are not fully developed, digestive enzymes and gastric acid secretion are insufficient, and the gut microbiota has not yet been established [8,9,10]. Concurrently, this transition is accompanied by changes in food, coupled with the stress of mother-child separation, which leads to disruption of the gut microbiota of weaned animals and a series of symptoms, such as decrease in appetite, poor digestion, diarrhea, poor resistance, and increased susceptibility to pathogenic microorganisms [11,12,13,14]. Compared to the pre-weaning stage, mammals experience significant changes in gut microbiota after weaning, after which it tends to stabilize. In Chongming white goats, the α diversities of gut and fecal microbiota significantly increase after weaning, and this change varies with different weaning ages [15]. Therefore, comparing and analyzing the changes in mammalian gut microbiota pre- and post-weaning can help mammals safely pass through the weaning period from the perspective of gut microbiota [16].

Weaning in blue foxes (*Alopex lagopus*) is a high-risk period of disease and death in the course of artificial feeding [17]. During this period, having intestinal flora that can maintain internal stability and intestinal health and resist foreign pathogen invasion is particularly important [18]. Therefore, the purpose of this study was to characterize the intestinal microflora of blue foxes before and after weaning using 16s rRNA-sequencing technology to and explore the similarity and variation between fecal microflora structure and intestinal microflora structure to provide a theoretical basis for future microbial therapy during weaning and non-invasive research on the intestinal microflora of blue foxes.

## 2. Materials and Methods

### 2.1. The Management of the Blue Foxes and Sample Collection

The protocol was reviewed and approved by the Laboratory Animal Welfare and Ethics Committee, Northeast Agricultural University.

In this study, 30 weaning blue foxes were used as experimental animals. The selection criteria of blue foxes were good health, close relationship, similarity in age, and no significant difference in body weight between individuals (*p* > 0.05). All blue foxes were weaned at 45 days of age. From this group, three healthy blue foxes each were selected at 45 days of age (the day of weaning) and 80 days of age and sacrificed via intravenous bloodletting after administration of anesthesia. The abdominal cavity was dissected, the intestinal segments were separated, and each intestinal segment was subjected to two-way ligation. The samples of duodenum, jejunum, ileum, cecum, colon, and rectal contents in a bacteria-free environment from blue foxes aged 45 and 80 days and feces from blue foxes aged 80 days were collected and placed in a 5 mL frozen storage tube after liquid nitrogen treatment and stored at −80 °C for 16S rRNA sequencing.

### 2.2. DNA Extraction and Polymerase Chain Reaction (PCR) Amplification

DNA from intestinal contents and feces of blue fox was extracted using the TGuide S96 magnetic bead fecal genomic DNA extraction kit (Tiangen Biochemical Technology, Beijing, Co., Ltd., DP812, Beijing, China). DNA quality and concentration were determined using a NanoDropND-1000 spectrophotometer (Thermo Fisher Scientific, Waltham, MA, USA) and 1% agarose gel electrophoresis.

The 16S rRNA fragment V3 and V4 regions of samples were amplified using primers (5′-ACTCCTACGGGAGGCAGCA-3′; 5′-GGACTACHVGGGTWTCT). PCR amplification in 25 µL reaction system included 5 µL of buffer solution (5×), 0.5 µL of FastPfu DNA polymerase (5 U/µL), 2 µL of dNTPs (2.5 µM), 0.5 µL of forward and reverse primers (10 µM), 2.5 µL of DNA template, and 14 µL of ddH_2_O. Reaction program: pre-denaturation at 95 °C for 5 min, 30 cycles (denaturation at 95 °C for 30 s, annealing at 50 °C for 30 s, extension at 72 °C for 40 s), and a final extension at 72 °C for 7 min. Finally, 1.8% agarose and an AxyPrep DNA Gel Extraction Kit were used to recover and purify the PCR products, respectively.

### 2.3. Illumina MiSeq Sequencing

The purified amplified fragments were prepared into a PE 2*300 library following the standard operating procedures of the Illumina Novaseq 6000 (Biomarker Technologies, Beijing, China).

The raw image data files obtained after the Illumina Novaseq sequencing platform were analyzed via base calling and transformed into the sequenced reads. The results were stored in the FASTQ file format, which contains the sequence information of the reads and their corresponding qualities.

### 2.4. Processing of Sequencing Data

Data preprocessing involved the following steps: (1) quality-filtering: the raw reads were filtered and identified, and primer sequences were removed using Trimmatic v0.33 software and cutadapt 1.9.1 software, respectively; (2) double-ended sequence splicing: the Usearch v10 software was used to splice the clean reads of each sample through overlap; (3) UCHIME v4.2 software was used to remove chimeras. Usearch [19] was used to cluster reads at a 97.0% similarity and obtain operational taxonomic units (OTUs). All sequences are divided into OTUs based on different levels of similarity.

### 2.5. Statistical Analysis

The species classification information corresponding to each feature were obtained using SILVA [20], Unite [21], Greengenes [22], NCBI [23], fungene [24], and MaarjAM [25] as reference databases for taxonomic annotation of feature sequences. Then, the community composition of each sample at various levels was statistically analyzed.

QIIME 2 software was used to generate species abundance tables at different taxonomic levels and to evaluate the alpha diversity index of the sample, and R language tools were used to draw community structure diagrams for each taxonomic level of the sample [26]. A *t*-test was used to evaluate the differences in alpha diversity index between different treatments.

## 3. Results

### 3.1. Evaluation of Sample Sequencing

**Figure 1:**
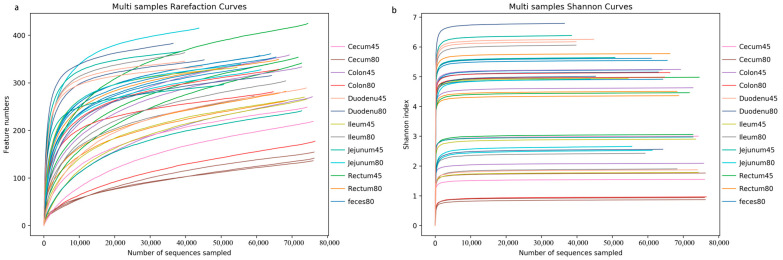
Presentation of sequencing depth of all samples. (**a**) Rarefaction curve, (**b**) Shannon curve.

The sequences were clustered into OTUs based on 97% sequence identity. The sequencing depth was assessed by plotting the rarefaction curve and a Shannon curve. As shown in Figure 1a, the rarefaction curve did not reach a plateau, while according to the Shannon curve, all samples entered the plateau phase (Figure 1b), indicating that at the current sequencing depth, this study could capture the vast majority of species in the samples, which met subsequent analysis requirements.

### 3.2. Difference of Intestinal Flora of Blue Fox before and after Weaning

#### 3.2.1. Sample Diversity Analysis

Shannon’s index, Simpson’s index, Chao1, and ACE were used to evaluate the alpha diversity of different intestinal segments of the blue foxes before and after weaning (Table 1). From the table, ACE index and Chao1 index of cecum before weaning was significantly and extremely significantly higher than that after weaning (*p* < 0.05; *p* < 0.01), and ACE index of cecum before weaning was significantly higher than ACE index of cecum after weaning (*p* < 0.05).

#### 3.2.2. Venn Diagram Analysis Based on Operational Taxonomic Units

**Figure 2:**
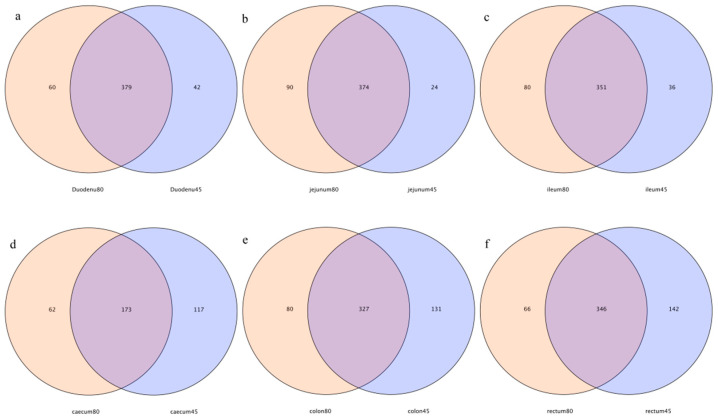
Operational-taxonomic-units-based Venn diagram analysis of blue fox intestinal flora before and after weaning; (**a**) duodenum, (**b**) jejunum, (**c**) ileum, (**d**) cecum, (**e**) colon, and (**f**) rectum of blue fox intestinal flora before and after weaning.

As shown in the Venn diagrams (Figure 2), 379, 374, 351, 173, 327, and 346 common OTUs were detected in the duodenum, jejunum, ileum, cecum, colon, and rectum of blue foxes before and after weaning, respectively. The specific OTUs of the duodenum, jejunum, ileum, cecum, colon, and rectum before and after weaning, were 42 vs. 60, 24 vs. 90, 36 vs. 80, 117 vs. 62, 113 vs. 80, and 142 vs. 66, respectively (Figure 2).

#### 3.2.3. Multi-Sample Comparative Analysis

**Figure 3:**
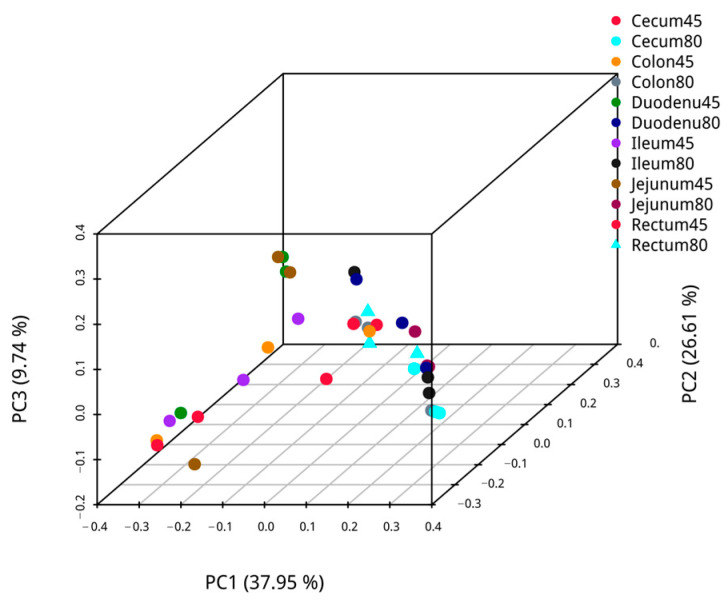
The similarity of intestinal flora of blue foxes before and after weaning.

Employing weighted UniFrac, principal coordinate analysis was performed on pre- and post-weaning intestinal samples of blue foxes, and the analysis results are presented in Figure 3. The first axis’s confidence of PCoA of microbiota *β* diversity was 39.39%, and the second axis’s confidence was 21.01% for the duodenal, and 42.68% and 17.28% for the jejunum. The confidence of PCoA analysis for microbial β-diversity of ileal microbiota was 39.43% in the first axis and 19.91% in the second axis, while those of cecum microbiota were 47.20% and 24.11%, respectively. Likewise, the confidence of the first axis and the second axis of β diversity PCoA analysis of colon microbiota were 46.98% and 31.11%, respectively, and those of the rectum were 51.28% for the first axis and 18.62% for the second axis. The PCoA shows that the intestinal communities of blue foxes were relatively dispersed before and after weaning; hence, the intestinal flora structures of blue foxes changed greatly before and after weaning.

#### 3.2.4. Structural Analysis of Intestinal Microbiota

**Figure 4:**
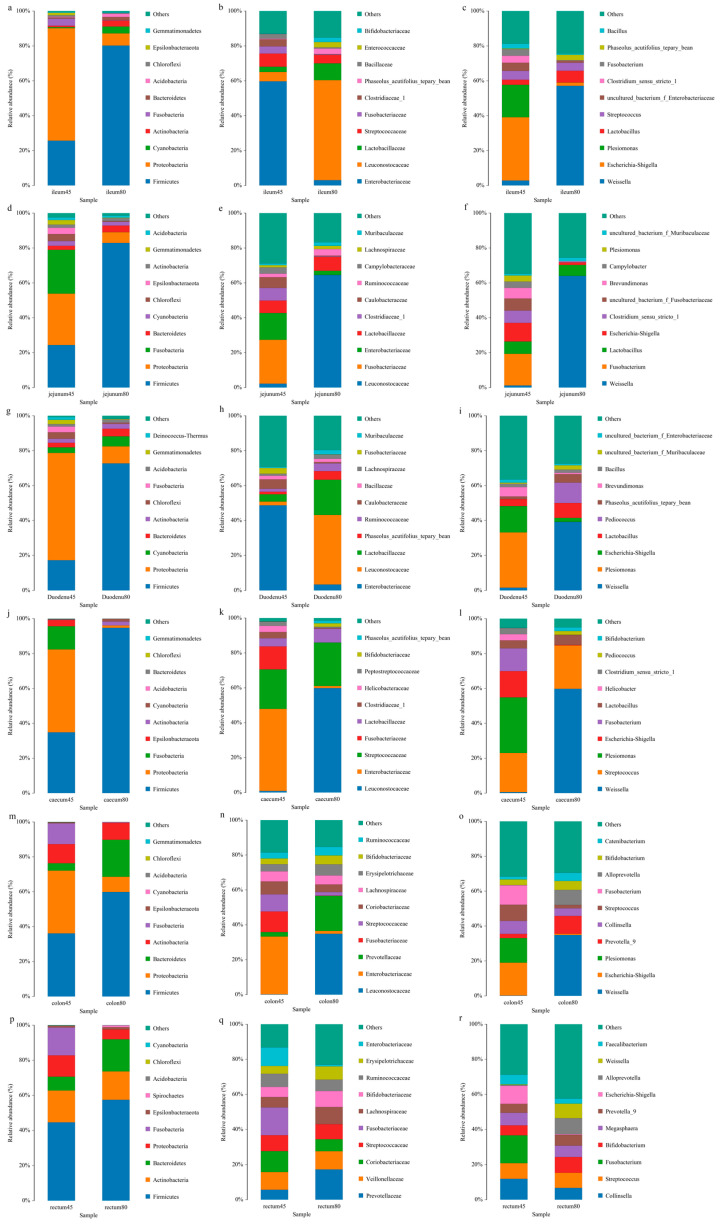
Relative abundance of intestinal flora at the levels of phylum, family, and genus of blue foxes before and after weaning. (**a**) ileum of 45 d and 80 d at the levels of phylum, (**b**) ileum of 45 d and 80 d at the levels of family, (**c**) ileum of 45 d and 80 d at the levels of genus, (**d**) jejunum of 45 d and 80 d at the levels of phylum, (**e**) jejunum of 45 d and 80 d at the levels of family, (**f**) jejunum of 45 d and 80 d at the levels of genus, (**g**) duodenum of 45 d and 80 d at the levels of phylum, (**h**) duodenum of 45 d and 80 d at the levels of family, (**i**) duodenum of 45 d and 80 d at the levels of genus, (**j**) caecum of 45 d and 80 d at the levels of phylum, (**k**) caecum of 45 d and 80 d at the levels of family, (**l**) caecum of 45 d and 80 d at the levels of genus, (**m**) colon of 45 d and 80 d at the levels of phylum, (**n**) colon of 45 d and 80 d at the levels of family, (**o**) colon of 45 d and 80 d at the levels of genus, (**p**) rectum of 45 d and 80 d at the levels of phylum, (**q**) rectum of 45 d and 80 d at the levels of family, (**r**) rectum of 45 d and 80 d at the levels of genus.

The relative abundances of intestinal flora at phylum levels of young foxes before and after weaning of young foxes are shown in Figure 4a. Compared with the pre-weaning stage, the abundances of duodenal and jejunal Firmicutes were increased (*p* < 0.05) significantly decreased (*p* < 0.01) significantly, respectively; the abundances of Proteobacteria and Cyanobacteria in the ileum were significantly increased (*p* < 0.05), while that of Bacteroidetes in the cecum was significantly reduced (*p* < 0.01), and the abundance of Acidobacterium in the colon and rectum was significantly increased (*p* < 0.05 and *p* < 0.01). In addition, before and after weaning, each intestinal segment of blue fox had different dominant flora. Firmicutes was the dominant microphylum in each intestinal segment of young fox before and after weaning. Moreover, Proteobacteria is the dominant microphylum in duodenum, jejunum, ileum, cecum and colon before weaning. Bacteroidetes also dominate the colon after weaning.

The relative abundances of intestinal microbiota at the family levels in blue foxes before and after weaning are shown in Figure 4b. Compared with pre-weaning, the abundance of Enterobacteriaceae and Leuconostocaceae in the jejunum reduced significantly after weaning (*p* < 0.01 and *p* < 0.05 respectively), while the abundance of Enterobacteriaceae in the ileum reduced significantly (*p* < 0.05) and that of *Phaseolus_ Acutifolius_ Team_ Beans* increased significantly (*p* < 0.05). The dominant family of bacteria in the duodenum, ileum, and colon pre-weaning was Enterobacaceae, while that post-weaning was Leuconostocaceae. The dominant families of bacteria in the jejunum pre-weaning were Enterobacteriaceae and Fusobacteriaceae, while that post-weaning was Leuconostocaceae. The dominant bacterial families in the cecum before weaning were Enterobacteriaceae and Streptococcaceae, while those after weaning were Leuconostocaceae and Streptococcaceae. The dominant family of bacteria in the rectum before weaning was Fusobacteriaceae, while that after weaning was Prevotellaceae.

The relative abundances of different genera of gut microbiota in blue foxes before and after weaning is shown in Figure 4c. Compared to pre-weaning, the abundance of *Shigella* in the duodenum and ileum decreased significantly (*p* < 0.05); the abundance of *Weissella* in the jejunum increased significantly (*p* < 0.05), while those of *Escherichia* and *Shigella* decreased significantly (*p* < 0.01). Concurrently, in the duodenum and cecum, the dominant genus of bacteria before weaning was *Plesiomonas*, and that after weaning was *Weissella*. The dominant genus of bacteria in the jejunum before weaning was *Fusobacterium*, and after weaning was *Weissella*. The dominant genus of bacteria in the ileum and colon before weaning were *Escherichia* and *Shigella*, and after weaning was *Weissella*. In the rectum, the dominant genus of bacteria before weaning was *Fusobacterium*, while after weaning, the distribution of the bacterial community was relatively uniform, with the main genera being *Weissella*, *Streptococcus*, *Bifidobacterium*, and *Alloprevotella*.

#### 3.2.5. LEfSe Species Difference Analysis

**Figure 5:**
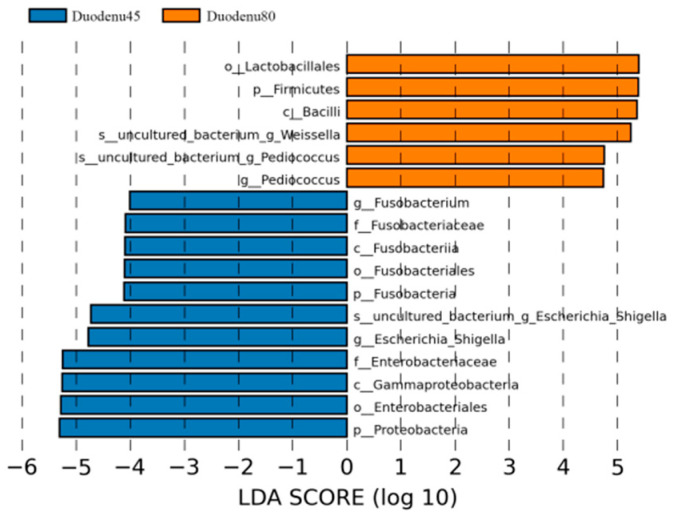
Linear discriminant analysis (LDA) analysis of duodenal intestinal flora of blue fox before and after weaning.

LEfSe analysis, based on the magnitude of the linear discriminant analysis (LDA) effects, is used to identify groups with significant differences in relative abundance in multi group comparisons. According to LDA > 4 as the screening criteria, the dominant bacterial communities in the duodenum of blue foxes before weaning were Proteobacteria, Fusobacteria, Firmicutes, Fusobacteria, Gammaproteobacteria, Fusobacteriales, Enterobacteriales, Fusobacteriaceae, Fusobacterium and Escherichia_Shigella; while the dominant microbiota in the duodenum of weaned blue foxes were Bacilli, Lactobacillales, and Uncultured_Bacterium_Weissella, and Uncultured_ Bacterium_Pediocucus (Figure 5).

**Figure 6:**
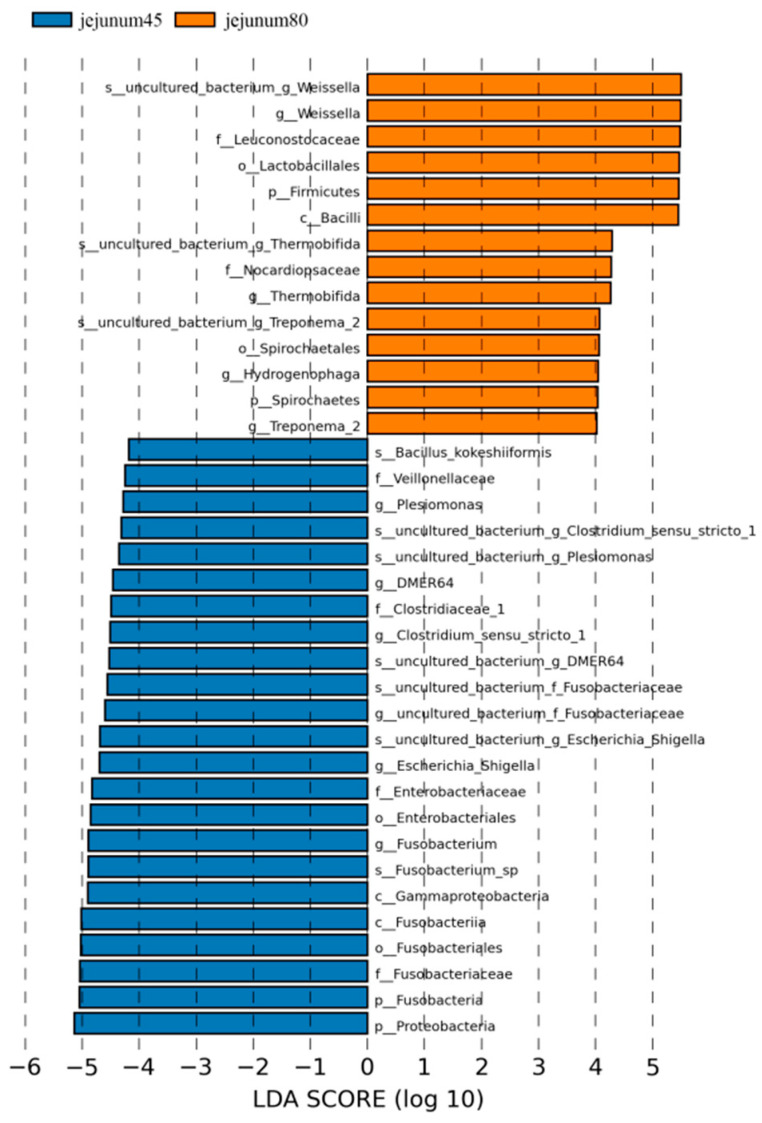
Linear discriminant analysis (LDA) of jejunal intestinal flora of blue fox before and after weaning.

Using LDA > 4 as the screening criteria, the dominant microbiota in jejunum before weaning were Proteobacteria, Gammaproteobacteria, Fusobacteria, Fusobacteriales, Enterobacteriales, Veillonellaceae, Clostridiaceae_1, Fusobacteriaceae, Enterobacteriaceae, Clostridium_Sensu_Stricto_1, Fusobacterium, Escherichia_ Shigella, Plesiomonas, Bacillus_Kokeshiiformis, and DMER64, while the dominant microbiota after weaning are Spirochaetes, Firmicutes, Bacilli, Lactobacillales, Spirochaetes, Nocardiopsaceae, Leuconostocaceae, Weissella, Thermobifida, and Treponema_2. Hydrogenophaga (Figure 6).

**Figure 7:**
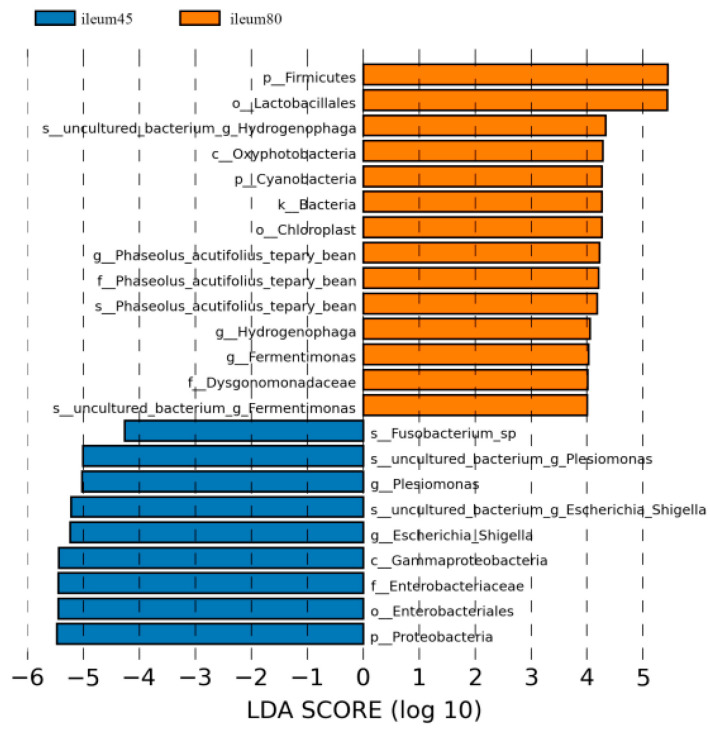
Linear discriminant analysis (LDA) of ileal intestinal flora of blue fox before and after weaning.

Using LDA > 4 as the screening criteria, the dominant microbiota in the ileum before weaning were Proteobacteria, Gammaproteobacteria, Enterobacteriales, Enterobacteriaceae, Fusobacterium, Plesiomonas, and Escherichia_Shigella, while the dominant microbiota in ileal after weaning were Firmicutes, Cyanobacteria, Oxyphotobacteria, Lactobacillales, Dysgonomonadaceae, and Phaseolus_Acutifolius_Team_Bean, Hydrogenophaga, Fermentimonas (Figure 7).

The LDA > 3 screening criteria could not screen any dominant microbiota in the cecum before and after weaning.

**Figure 8:**
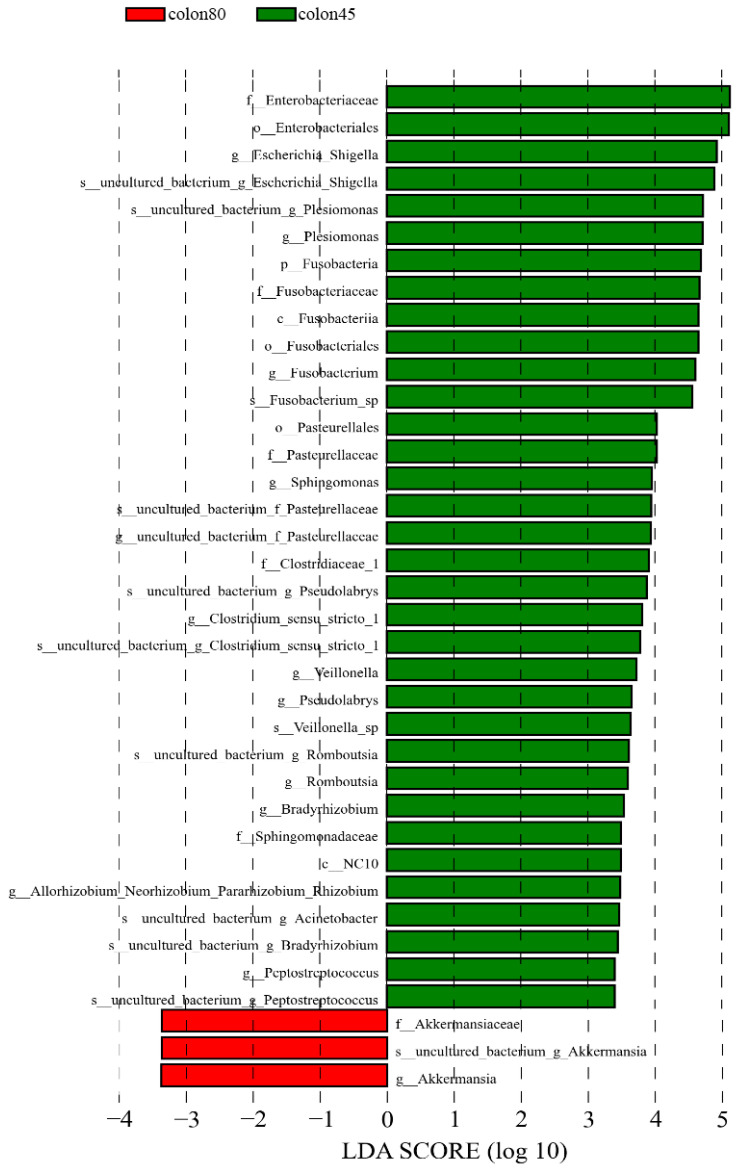
Linear discriminant analysis (LDA) of colonic intestinal flora of blue fox before and after weaning.

The LDA > 3.4 was used as the screening criteria, and the dominant microbiota in the pre weaning colon were Fusobacteria, Fusobacteria, NC10, Enterobacteriales, Fusobacteriales, Pasturellales, Enterobacteriaceae, Fusobacteriaceae, Pasturellaceae, and Clostridiaceae_1. Sphingomonadaceae, Fusobacterium, Escherichia_Shigella, Plesiomonas, Sphingomonas, Pseudolabs, Clostridium_Sensu_Stricto_1. Veillonella, Romboutsia, Bradyrhizobium, Peptostreptococcus, Uncultured_Bacterium_Acinetobacter, Allorhozobium_Neorhobium_Pararhobium_Rhizobium, while the dominant microbiota after weaning were Akkermansiaceae and Akkermansia (Figure 8).

**Figure 9:**
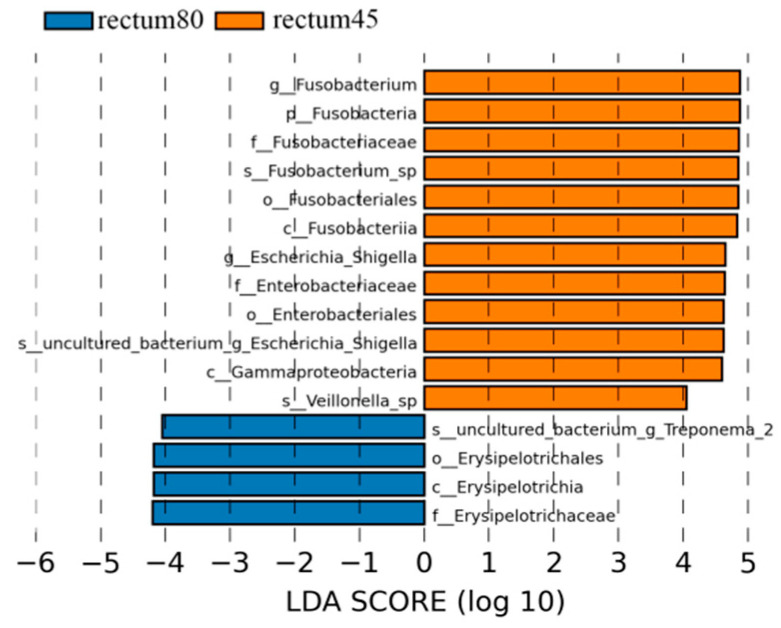
Linear discriminant analysis (LDA) analysis of rectal intestinal flora in blue fox before and after weaning.

According to the LDA > 4 screening criteria, the dominant microbial communities in the rectum before weaning were Fusobacteria, Gammaproteobacteria, Fusobacteria, Fusobacteriales, Enterobacteriales, Enterobacteriaceae, Fusobacteriaceae, Fusobacterium, Escherichia_Shigella, while the dominant microbiota after weaning were Erysipelotirichia, Erysipelotirichales, Erysipelotirichaceae, and Treponema_2 (Figure 9).

### 3.3. Analysis of Bacterial Flora Structure Difference between Fecal and Intestinal Contents

The structural differences between intestinal contents and fecal microbiota were analyzed using samples of intestinal contents and feces from 80-day-old blue foxes.

#### 3.3.1. Sample Diversity Analysis of Fecal and Intestinal Contents

Table 2 shows that the fecal ACE index, Chao1, and Shannon indexes were significantly higher than those of the cecum (*p* < 0.01), while the duodenal and ileal ACE indices were significantly higher than those of the cecum (*p* < 0.05). The duodenal ACE and Chao1 indexes were significantly higher than those of the cecum (*p* < 0.05). The ACE index of the ileum was significantly higher than that of the cecum (*p* < 0.05), The Shannon index of the rectum was significantly higher than that of the cecum (*p* < 0.05).

#### 3.3.2. Venn Diagram Analysis Based on Operational Taxonomic Units

**Figure 10:**
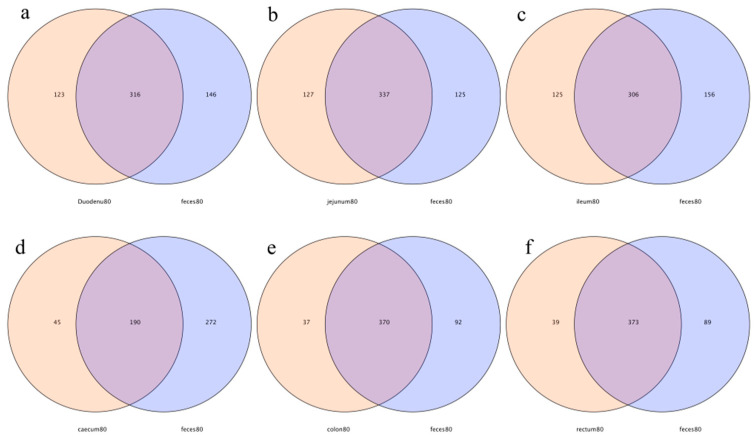
Operational-taxonomic-units-based Venn diagram analysis of contents in different intestinal segments and fecal flora; (**a**) duodenum vs. feces, (**b**) jejunum vs. feces, (**c**) ileum vs. feces, (**d**) cecum vs. feces, (**e**) colon vs. feces, and (**f**) rectum vs. feces.

In this study, 316, 337, 306, 190, 370, and 373 OTUs from the feces were shared by the duodenum, jejunum, ileum, cecum, colon, and rectum, respectively. The number of specific OTUs of feces and the duodenum, feces and the jejunum, feces and the ileum, feces and the cecum, feces and the colon, and feces and the rectum were 146 and 123, 125 and 127, 156 and 125, 272 and 45, 92 and 37, and 89 and 39, respectively. The number of specific OTUs of the large intestine was less than that of the small intestine, indicating that the OTUs’ compositions of feces and the large intestine were more similar (Figure 10).

#### 3.3.3. Multi-Sample Comparative Analysis

**Figure 11:**
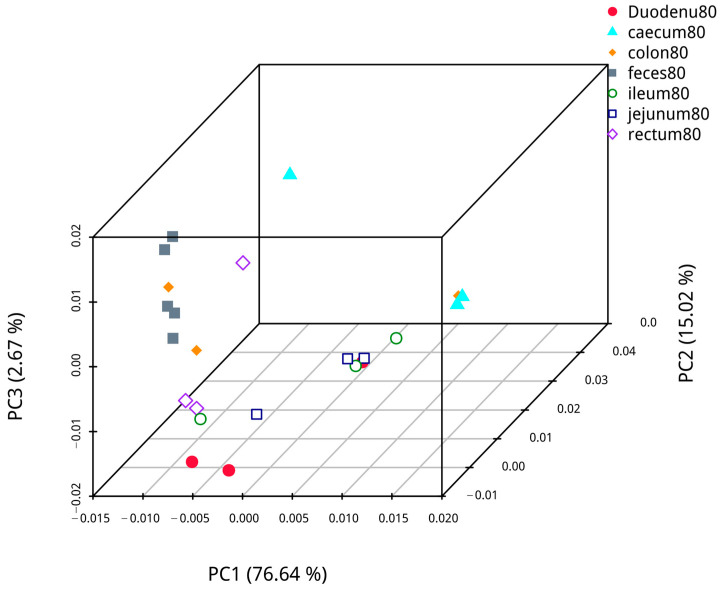
The similarity of contents in different intestinal segments and fecal flora.

Based on abundance information of the OTUs and species annotated results of all samples, PCoA based on weighted UniFrac was performed to compare the bacterial dominance of fecal samples with samples from different intestinal segments of blue foxes. The analysis results are shown in Figure 11. The first axis of confidence in fecal and duodenal microflora analysis was 74.98%, and the credibility of the second axis was 12.43%; analysis of fecal and jejunal microflora revealed that the confidence of the first axis was 88.73%, and that of the second axis was 5.46%. Analysis of fecal and ileal microflora showed that the confidence of the first axis was 87.58%, and that of the second axis was 5.59%. Analysis of fecal and cecal microflora showed that the confidence of the first axis was 70.56%, and that of the second axis was 26.51%. Analysis of the fecal and colon microflora showed that the confidence of the first axis was 87.59%, and the confidence of the second axis was 6.87%. Analysis of the fecal and rectal microflora revealed that that the confidence of the first axis was 40.07%, and that of the second axis was 22.97%. Hence, the samples of each intestinal segment were different, while the distance between colon, rectal, and fecal samples was similar, indicating that fecal samples are more similar to the samples from the colon and rectum than to other intestinal segments.

#### 3.3.4. UPGMA Cluster Analysis

**Figure 12:**
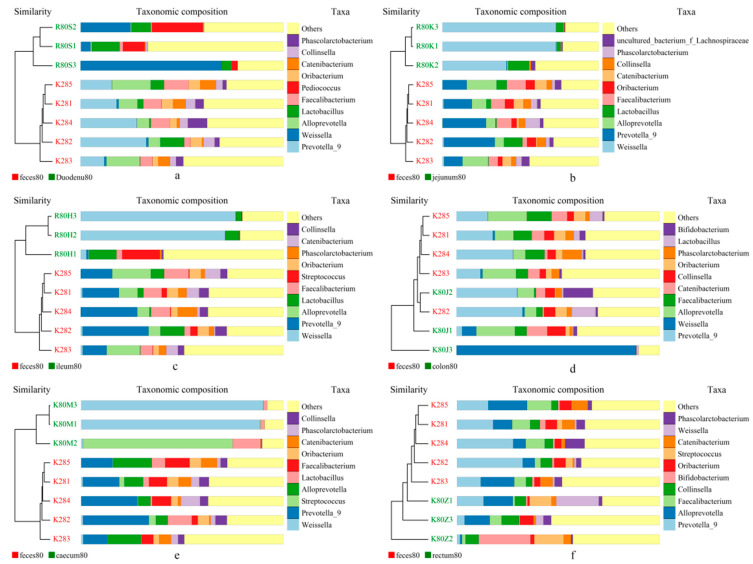
UPGMA clustering tree based on weighted UniFrac distance. (**a**) feces vs. duodenum; (**b**) feces vs. Jejunum; (**c**) feces vs. Ileum; (**d**) feces vs. colon; (**e**) feces vs. caecum, and (**f**) feces vs. rectum.

Two closest samples were clustered together to form a new node. Then, the average distance between one “sample” and the other samples was calculated, and two similar samples were clustered. Finally, all the samples were clustered to form a clustering tree for further analysis. As shown in Figure 12, the analysis described here for the flora composition of stool and each intestinal segment at the generic level revealed two distinct clusters—stool samples and intestinal samples—indicating that the bacterial community in the stool and each intestinal segment were significantly different. The UPGMA clustering results were similar to those of PCA analysis.

#### 3.3.5. Significance Test of Differences in Community Structure of Feces and Various Intestinal Contents

Anoism analysis is a non-parametric test based on the rank of Unweighted_unifrac distance values to determine whether the inter-group differences are greater than the intra-group differences. The R-value was between −1 and 1 and was >0 if the difference between stool and intestine groups was greater than the intra-group difference. If the difference between fecal and intestinal groups was less than the difference within groups, R-value was <0, and if the difference between or within groups was significant, then *p* was <0.05. According to Table 3, R-value > 0 indicates that the difference between feces and different intestinal microbial groups is greater than the intra-group differences. *p*-value < 0.05 indicates that the difference between feces and different intestinal microbial groups was significant. Colon and rectal contents exhibited microbiota structure closer to that of the feces.

#### 3.3.6. LEfSe Species Difference Analysis

**Figure 13:**
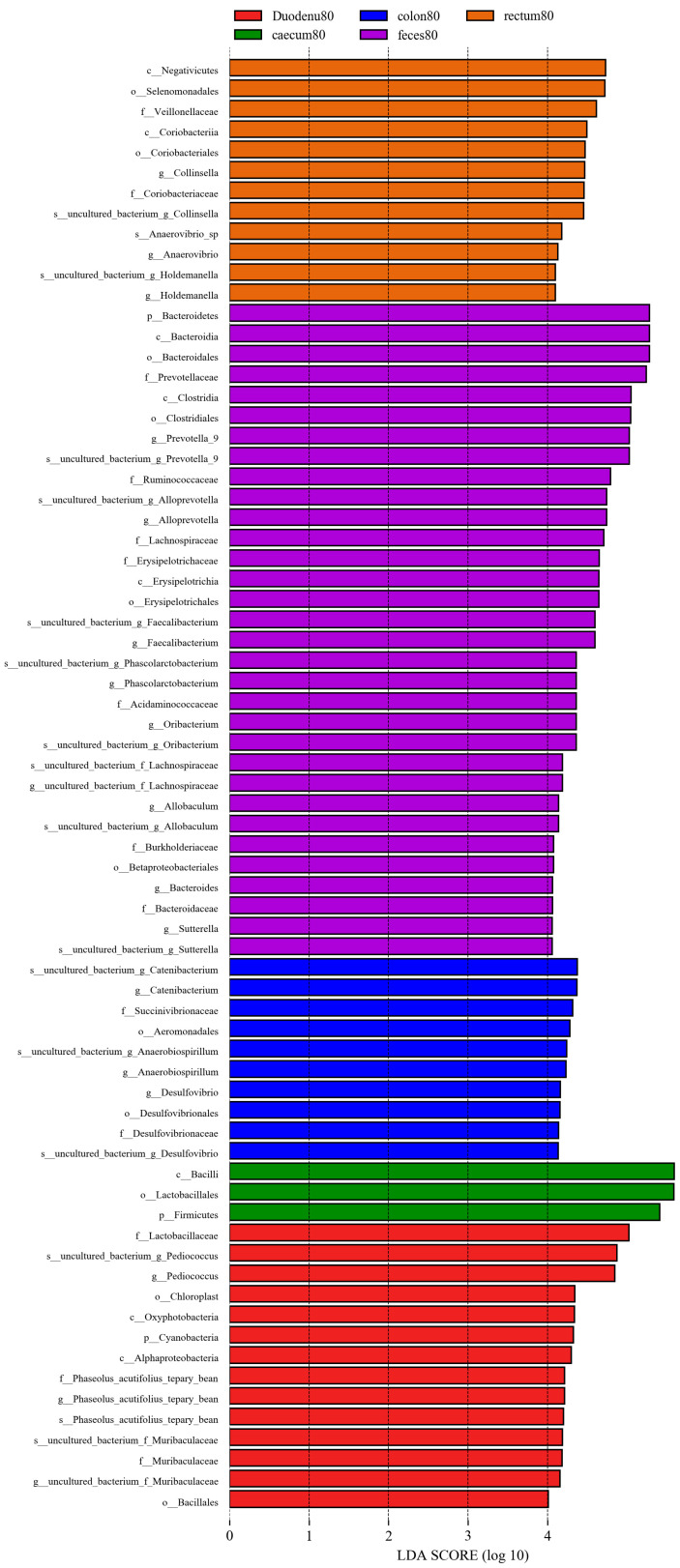

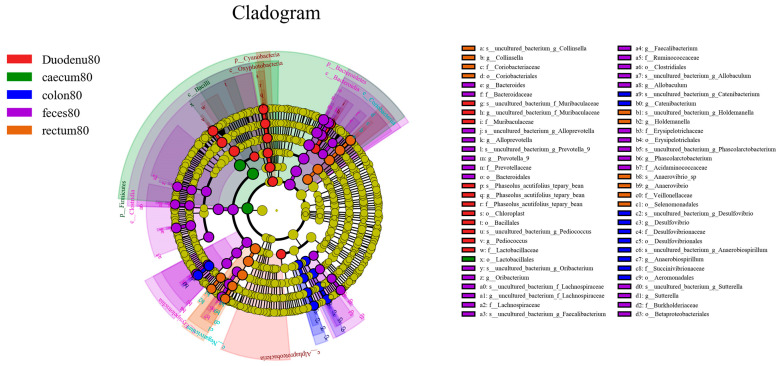
LEfSe analysis of differential species between different sample groups.

As shown in Figure 13, considering LDA > 4 as the screening criterion, 72 bacteria showed statistically significant differences in abundance between feces and various intestinal segments. Among these, the bacterial communities significantly higher in feces than in various intestinal segments were Bacteroidetes, Bacteroidia, Clostridia, Erysipelotrichia, Bacteroidales, Clostridiales, Erysipelotrichales, Betaprotebacteria, Bacteroidaceae, Prevotellaceae, Ruminococcaceae, Lachnospiraceae, Erysipelotrichaceae, Acidaminococcaceae, Burkholderiaceae, Bacteroides, Prevotella-9, Alloprevotella, Faecalibacterium, Phascolarctobacter, Allobaculum, and Sutterella; the bacterial communities in the duodenum, which are significantly higher than those in feces and other intestinal segments, were Cyanobacteria, Acidobacteria, Oxyphotobacteria, Alphaproteobacteria, Bacilliales, Lactobacilliaceae, f_Phaseolus_acutifolius_tepary_bean, Muribaculaceae, Pediococcus, g_Phaseolus_acutifolius_tepary-bean, and Muribaculaceae. In the cecum, bacterial communities significantly higher than those in the feces and other intestinal segments were Firmicutes, Bacilli, and Lactobacilliales. The bacterial communities that were significantly higher in the colon than those in feces and other intestinal segments were Aeromonadales, Desulfovibrionales, Succinivibrionaceae, Desulfovibrionaceae, Catenibacterium, Anaerobiospirillum, and Desulfovibrio; the bacterial communities significantly higher in the rectum than in the feces and other intestinal segments were Negativicutes, Coriobacteriia, Selenomonadales, Coriobacteriales, Veillonellaceae, Coriobacteriaceae, Holdemanella, Anaerovibrio, and Collinsella.

## 4. Discussion

The gut microbiota of animals changes with age and diet [27]. Mammals inevitably go through the weaning stage, accompanied by aging and changes in diet. Our study results indicate that the intestinal flora of blue fox is constantly changing—thus complicating the establishment of the bacterial community with the change in age and diet—and tends to be complicated and diversified. At each growth stage, characteristic or dominant flora are formed to play corresponding roles. The study of intestinal flora of blue fox before and after weaning reveals the dynamic succession rule of intestinal flora and helps us strengthen the understanding of intestinal microecology of blue fox to provide a theoretical basis for improving the feeding and management of blue foxes during the weaning period.

The study results show that the distribution of different intestine segments of blue foxes before and after weaning was relatively dispersed, indicating that the community composition of the samples is less similar and that the difference is significantly large. Therefore, the microflora structure of each intestinal segment changes significantly before and after weaning. The α diversity index analysis showed that ACE, Chao1, Shannon, and Simpson indices fluctuated from duodenum to rectum before and after weaning. In addition, the ACE index and Chao1 index of the cecum were significantly higher before weaning than after weaning. This may be because stress such as weaning and litter division caused the decrease in bacterial diversity in the cecum and rectum.

Chen et al. reported that Firmicutes, Bacteroidetes, Actinobacteria, Proteobacteria, and Fusobacteria were the dominant bacteria groups in the intestinal tract of the blue fox, accounting for 99.94% of the total bacterial population [28]. This study shows that at the gate level, the intestinal dominant bacteria group of weaning blue foxes changed from Firmicutes and Proteobacteria to only Firmicutes before and after weaning. The dominant bacteria in the cecums and the colons of weaned blue foxes changed from Firmicutes and Proteobacteria to Firmicutes before and after weaning. The dominant intestinal bacteria in the colons of weaned blue foxes changed from Firmicutes and Proteobacteria to Firmicutes (colon) and Firmicutes and Bacteroidetes (cecum) before and after weaning. The intestinal flora of the rectum were predominantly Firmicutes before as well as after weaning. The primary reason for the difference in the results of the two studies may be the different ages of the experimental blue foxes.

Recent research has found that bacterial communities in the intestine are associated with obesity [29]. Among these, Firmicutes are associated with the digestion of fat and protein [30]. With the increase in the ratio of Firmicutes/Bacteroidetes in the intestinal tract of obese people, the amount of energy taken in by the body increases [30,31]. Therefore, the absorption capacity of fat and protein in blue fox small intestines after weaning may have been stronger than that before weaning in our study. In our study, the content of Proteobacteria in each intestine segment at the late weaning stage was lower than that before weaning. Current research has also revealed that the decrease in the content of Proteobacteria is correlated with cage separation, weaning stress [9], and age [32].

At the genus level, before weaning, the dominant bacteria was Plesiomonas in the duodenum and cecum, Fusobacterium in the jejunum, and Escherichia-Shigella in the ileum and colon. The dominant bacteria in rectum and jejunum were the same, being Fusobacterium. However, after weaning, the dominant bacteria in duodenum, jejunum, ileum, cecum, and colon were different from those in rectum—Alloprevotella—while other intestinal segments had Weissella predominantly. Weissella can produce CO_2_, ethanol, and/or acetic acid by fermenting glucose [33], and a causal relationship exists between changes in gut flora and increased acetic acid production [34]. The increase in acetic acid leads to the increased dietary intake, which leads to the positive feedback effect of insulin resistance and obesity, which is the same condition as the gradual increase in food intake and fat intake during weaning of blue foxes. Weissella, a member of the lactic acid bacteria family, has certain probiotic properties, such as anti-fungal, prolactobacillus proliferation, etc. [35].

When the young blue fox enters the post-weaning period, its nutritional structure changes from the milk of the mother to feed, and the young fox stops taking in the antibacterial substances present in the milk of the mother fox; thus, the bacteria in the intestine begin to multiply in large numbers and gradually colonize the intestine. However, the influence of cage separation, weaning stress, companion separation, and other factors also affects the intestinal flora of the blue fox and also causes fluctuation in the development and colonization of the gut microbiota in the weaning period. In pigs, the intestinal microflora changes continuously from birth to lactation, and the abundance of microflora gradually increases. During lactation, the intestinal microflora of piglets tends to be stable, and after the weaning period, a relatively stable microecology is formed through long-term adjustment of the microflora structure [36]. By comparing the six intestinal segments before and after weaning, the intestinal microbial communities of young foxes could be grouped into one type before and after weaning, indicating that the microbial communities of each intestinal segment of blue foxes had their own structural characteristics. In addition, the structure and abundance of the intestinal flora of blue foxes changed with the change in age and dietary intake before and after weaning.

Physiologically, there exists a clear functional boundary between the large intestine and the small intestine. In this study, there were significant differences in the types and distribution of intestinal microbes in different parts of the intestine, and the distribution pattern was basically consistent with those reported in previous studies [37,38]. At the same time, due to species diversity and the specificity of the gut microbiome distribution in the host, the gut microbiomes of each host may vary slightly [33,39,40,41,42].

In the study of gut microbiota in humans and animals feces are usually used instead of gut microbiota. Although PCA analysis in our study showed high similarity between the fecal microbiota and that of the colon and rectum of blue foxes before and after weaning, UPGMA analysis revealed that at the genus level, each gut and fecal microbiota clustered into a distinct group. Further analysis using Anosim yielded the same results, indicating significant differences between feces and the microbial population in different sections of the intestine. Nevertheless, compared to the duodenum and cecum, the microbiota in the colon and rectum were closer in structure to the fecal microbiota. This may be because the contents of the colon and rectum are relatively close to that of feces. LEfSe analysis of fecal and intestinal microbiota showed significant differences between fecal microbiota such as Bacteroides and Ruminococcus at the levels of class, order, family, and genus in different intestinal segments.

In this study, significant individual differences that may have been caused by factors such as weaning stress and dietary changes, which may be the cause of inter group differences. Therefore, the similarity between fecal and intestinal microbiota needs to be further studied by expanding the sample size. Based on the results of this study, which show that the microbiota in the colon and rectum are more like those of fecal microbiota structure, feces is more likely to represent the microbiota of the colon and the rectum. Nonetheless, other methods should still be chosen for the assessment of microbiota of other intestinal segments.

## 5. Conclusions

To summarize, the microbial species and distribution characteristics in different intestinal segments of blue fox were different before and after weaning. Except for the rectum, the dominant flora of each intestinal segment changed significantly before and after weaning. The fecal flora of weaned young foxes did not represent the whole intestinal flora but approximately represented the intestinal flora of colon and rectum.

## Figures and Tables

**Table 1:** The alpha diversity of the microbial contents from different intestinal segments of blue fox before and after weaning.

Items		ACE Index	Chao1 Index	Simpson Index	Shannon Index
Duodenum	45 d	341.32 ± 14.79	343.53 ± 13.37	0.81 ± 0.26	4.77 ± 2.51
	80 d	364.47 ± 33.69	373.41 ± 36.32	0.79 ± 0.24	4.79 ± 2.12
Jejunum	45 d	323.84 ± 46.86	324.25 ± 47.27	0.90 ± 0.09	5.00 ± 1.79
	80 d	383.55 ± 41.35	390.21 ± 52.82	0.59 ± 0.20	3.37 ± 1.34
Ileum	45 d	327.17 ± 15.00	331.82 ± 15.96	0.74 ± 0.16	3.21 ± 1.60
	80 d	354.07 ± 30.32	367.79 ± 47.15	0.61 ± 0.29	3.46 ± 2.26
Cecum	45 d	283.00 ± 7.48 ^a^	286.53 ± 7.42 ^A^	0.64 ± 0.15	2.28 ± 1.02
	80 d	206.04 ± 30.87 ^b^	183.06 ± 10.59 ^B^	0.28 ± 0.14	1.19 ± 0.50
Colon	45 d	371.03 ± 34.30	369.20 ± 28.98	0.84 ± 0.13	3.99 ± 1.67
	80 d	314.35 ± 52.45	323.38 ± 48.91	0.69 ± 0.41	3.69 ± 2.37
Rectum	45 d	441.77 ± 25.44 ^a^	452.93 ± 32.70	0.85 ± 0.11	4.17 ± 0.99
	80 d	348.79 ± 34.39 ^b^	360.60 ± 54.82	0.93 ± 0.04	4.88 ± 0.78

^a,b^ In the same intestinal segment at different time, values with different lowercase superscripts indicate a significant difference (*p* < 0.05); ^A,B^ In the same intestinal segment at different time, values with different lowercase superscripts indicate a extremely significant difference (*p* < 0.01).

**Table 2:** Alpha diversity index of different intestinal segments and feces.

Items	ACE Index	Chao1 Index	Simpson Index	Shannon Index
feces	392.65 ± 11.82 ^Aab^	406.49 ± 23.06 ^Aab^	0.93 ± 0.02	5.29 ± 0.29 ^Aab^
duodenum	364.47 ± 33.69 ^Aba^	373.41 ± 36.32 ^Aba^	0.79 ± 0.24	4.79 ± 2.12 ^Abab^
jejunum	383.55 ± 41.35 ^Abab^	390.21 ± 52.82 ^Abab^	0.59 ± 0.20	3.37 ± 1.34 ^Abab^
ileum	354.07 ± 30.32 ^Aba^	367.79 ± 47.15 ^Abab^	0.61 ± 0.29	3.46 ± 2.26 ^Abab^
cecum	206.04 ± 30.87 ^Bb^	183.06 ± 10.59 ^Bb^	0.28 ± 0.14	1.19 ± 0.50 ^Bb^
colon	314.35 ± 52.45 ^Abab^	323.38 ± 48.91 ^Abab^	0.69 ± 0.41	3.69 ± 2.37 ^Abab^
rectum	348.79 ± 34.39 ^Abab^	360.60 ± 54.82 ^Abab^	0.93 ± 0.04	4.88 ± 0.78 ^Aba^

^a,b^ In the same column, values with different lowercase superscripts indicate a significant difference (*p* < 0.05); ^A,B^ In the same column, values with different lowercase superscripts indicate a extremely significant difference (*p* < 0.01).

**Table 3:** Anosim analysis of feces and different intestinal microbial groups.

Items	R-Value	*p*-Value
Duodenum	1.000	0.017
Jejunum	1.000	0.018
Ileum	1.000	0.015
Cecum	1.000	0.020
Colon	0.744	0.018
Rectum	0.713	0.017

## Data Availability

The data presented in this study are available on request. These data are not publicly available to preserve the data privacy of the commercial farm.
